# The EU-AIMS Longitudinal European Autism Project (LEAP): clinical characterisation

**DOI:** 10.1186/s13229-017-0145-9

**Published:** 2017-06-23

**Authors:** Tony Charman, Eva Loth, Julian Tillmann, Daisy Crawley, Caroline Wooldridge, David Goyard, Jumana Ahmad, Bonnie Auyeung, Sara Ambrosino, Tobias Banaschewski, Simon Baron-Cohen, Sarah Baumeister, Christian Beckmann, Sven Bölte, Thomas Bourgeron, Carsten Bours, Michael Brammer, Daniel Brandeis, Claudia Brogna, Yvette de Bruijn, Bhismadev Chakrabarti, Ineke Cornelissen, Flavio Dell’ Acqua, Guillaume Dumas, Sarah Durston, Christine Ecker, Jessica Faulkner, Vincent Frouin, Pilar Garcés, Lindsay Ham, Hannah Hayward, Joerg Hipp, Rosemary J. Holt, Johan Isaksson, Mark H. Johnson, Emily J. H. Jones, Prantik Kundu, Meng-Chuan Lai, Xavier Liogier D’ardhuy, Michael V. Lombardo, David J Lythgoe, René Mandl, Luke Mason, Andreas Meyer-Lindenberg, Carolin Moessnang, Nico Mueller, Laurence O’Dwyer, Marianne Oldehinkel, Bob Oranje, Gahan Pandina, Antonio M. Persico, Barbara Ruggeri, Amber N. V. Ruigrok, Jessica Sabet, Roberto Sacco, Antonia San Jóse Cáceres, Emily Simonoff, Roberto Toro, Heike Tost, Jack Waldman, Steve C. R. Williams, Marcel P. Zwiers, Will Spooren, Declan G. M. Murphy, Jan K. Buitelaar

**Affiliations:** 1 Department of Psychology, Institute of Psychiatry, Psychology and Neuroscience, King’s College London, De Crespigny Park, Denmark Hill, London, SE5 8AF UK; 2 Sackler Institute for Translational Neurodevelopment, Institute of Psychiatry, Psychology and Neuroscience, King’s College London, De Crespigny Park, Denmark Hill, London, SE5 8AF UK; 3 Department of Forensic and Neurodevelopmental Sciences, Institute of Psychiatry, Psychology and Neuroscience, King’s College London, De Crespigny Park, Denmark Hill, London, SE5 8AF UK; 4 Department of Neuroimaging, Institute of Psychiatry, Psychology and Neuroscience, King’s College London, De Crespigny Park, Denmark Hill, London, SE5 8AF UK; 5 Neurospin Centre CEA, Saclay, 91191 Gif sur Yvette France; 6 Autism Research Centre, Department of Psychiatry, University of Cambridge, Douglas House, 18b Trumpington Road, Cambridge, CB2 8AH UK; 7 The School of Philosophy, Psychology, and Language Sciences, University of Edinburgh, Dugald Stewart Building, 3 Charles Street, Edinburgh, EH8 9AD UK; 8 Department of Psychiatry, Brain Center Rudolf Magnus, University Medical Center Utrecht, Universiteitsweg 100, 3584 CG, Utrecht, The Netherlands; 9 Child and Adolescent Psychiatry, Central Institute of Mental Health, University of Heidelberg, Medical Faculty Mannheim, J5, 68159 Mannheim, Germany; 10 Donders Institute for Brain, Cognition and Behaviour, Radboud University Nijmegen Medical Centre, Kapittelweg 29, 6525 EN Nijmegen, The Netherlands; 11 Center for Neurodevelopmental Disorders at Karolinska Institutet (KIND), Stockholm, Sweden; 12 Child and Adolescent Psychiatry, Center of Psychiatry Research, Stockholm County Council, Stockholm, Sweden; 13 Institut Pasteur, Human Genetics and Cognitive Functions Unit, 25 Rue du Docteur Roux, Paris, Cedex 15 France; 14 University Campus Bio-Medico, Via Álvaro del Portillo, 21, Rome, Italy; 15 School of Psychology and Clinical Language Sciences, University of Reading, Whiteknights, Reading, RG6 6AL UK; 16 Department of Child and Adolescent Psychiatry, Psychosomatics and Psychotherapy, University Hospital Frankfurt am Main, Goethe University, Deutschordenstrasse 50, 60528 Frankfurt, Germany; 17 Roche Pharma Research and Early Development, Neuroscience, Ophthalmology and Rare Diseases, Roche Innovation Center Basel, Grenzacherstrasse 124, B.001 N.667, CH-4070 Basel, Switzerland; 18 Regulatory Affairs, Pharmaceutical Development, F. Hoffmann-La Roche Pharmaceuticals, Grenzacherstrasse 124, CH-4070 Basel, Switzerland; 19 Department of Neuroscience, Uppsala University, Uppsala, Sweden; 20 Centre for Brain and Cognitive Development, Birkbeck, University of London, 32 Torrington Square, London, WC1E 7JL UK; 21 Department of Radiology, Icahn School of Medicine at Mount Sinai, NY, USA; 22 Child and Youth Mental Health Collaborative, Centre for Addiction and Mental Health and The Hospital for Sick Children, Department of Psychiatry, University of Toronto, 80 Workman Way, Toronto, ON M6J 1H4 Canada; 23 Center for Applied Neuroscience, Department of Psychology, University of Cyprus, PO Box 20537, 1678 Nicosia, Cyprus; 24 Department of Psychiatry and Psychotherapy, Central Institute of Mental Health, Medical Faculty Mannheim, University of Heidelberg, 68159 Mannheim, Germany; 25 Janssen Research & Development, 1125 Trenton Harbourton Road, Titusville, NJ 08560 USA; 26 Child and Adolescent Neuropsychiatry Unit, “Gaetano Martino” University Hospital, University of Messina, via Consolare Valeria 1, I-98125 Messina, Italy; 27 Social, Genetic and Developmental Psychiatry Centre, Institute of Psychiatry, Psychology and Neuroscience, King’s College London, Denmark Hill, London, UK; 28 Department of Child and Adolescent Psychiatry, Institute of Psychology, Psychiatry and Neuroscience, King’s College London, De Crespigny Park, Denmark Hill, London, SE5 8LF UK; 29 Roche Pharmaceutical Research and Early Development, NORD Discovery and Translational Area, Roche Innovation Center Basel, Grenzacherstrasse 124, CH-4070 Basel, Switzerland

**Keywords:** Autism, Autism spectrum disorder, Phenotype, Behaviours, Heterogeneity, Sex, Age, IQ

## Abstract

**Background:**

The EU-AIMS Longitudinal European Autism Project (LEAP) is to date the largest multi-centre, multi-disciplinary observational study on biomarkers for autism spectrum disorder (ASD). The current paper describes the clinical characteristics of the LEAP cohort and examines age, sex and IQ differences in ASD core symptoms and common co-occurring psychiatric symptoms. A companion paper describes the overall design and experimental protocol and outlines the strategy to identify stratification biomarkers.

**Methods:**

From six research centres in four European countries, we recruited 437 children and adults with ASD and 300 controls between the ages of 6 and 30 years with IQs varying between 50 and 148. We conducted in-depth clinical characterisation including a wide range of observational, interview and questionnaire measures of the ASD phenotype, as well as co-occurring psychiatric symptoms.

**Results:**

The cohort showed heterogeneity in ASD symptom presentation, with only minimal to moderate site differences on core clinical and cognitive measures. On both parent-report interview and questionnaire measures, ASD symptom severity was lower in adults compared to children and adolescents. The precise pattern of differences varied across measures, but there was some evidence of both lower social symptoms and lower repetitive behaviour severity in adults. Males had higher ASD symptom scores than females on clinician-rated and parent interview diagnostic measures but not on parent-reported dimensional measures of ASD symptoms. In contrast, self-reported ASD symptom severity was higher in adults compared to adolescents, and in adult females compared to males. Higher scores on ASD symptom measures were moderately associated with lower IQ. Both inattentive and hyperactive/impulsive ADHD symptoms were lower in adults than in children and adolescents, and males with ASD had higher levels of inattentive and hyperactive/impulsive ADHD symptoms than females.

**Conclusions:**

The established phenotypic heterogeneity in ASD is well captured in the LEAP cohort. Variation both in core ASD symptom severity and in commonly co-occurring psychiatric symptoms were systematically associated with sex, age and IQ. The pattern of ASD symptom differences with age and sex also varied by whether these were clinician ratings or parent- or self-reported which has important implications for establishing stratification biomarkers and for their potential use as outcome measures in clinical trials.

**Electronic supplementary material:**

The online version of this article (doi:10.1186/s13229-017-0145-9) contains supplementary material, which is available to authorized users.

## Background

### Heterogeneity is a core feature of the ASD phenotype

Autism spectrum disorder (ASD) is a common neurodevelopmental disorder, affecting ~1% of children and adults [1–4]. The core characteristics are impairments in social communication abilities, the presence of rigid, repetitive and stereotyped behaviours, and atypical sensory responses (DSM-5; [5]). However, there is wide heterogeneity in clinical presentation, both in terms of symptom profiles and severity (hence the use of the term ‘spectrum’; [6]) and levels of intellectual and functional communication ability. Commonly associated conditions range from psychiatric symptoms, such as anxiety disorders and attention-deficit/hyperactivity disorder (ADHD) [7] to medical conditions including epilepsy and gastrointestinal abnormalities [8]. Heterogeneity is present both *between* individuals who fulfil the diagnostic criteria and *within* individuals across development [9, 10]. Decomposing this heterogeneity may get us closer to more precise inferences about which subsets of individuals are best characterised by different cognitive theories of ASD [11]. Wide variability is also present at the level of aetiological mechanisms. Common genetic variants of small effect size are thought to accumulate and contribute towards enhanced risk, implicating a diverse range of biological pathways. Similarly, some rare genetic variants found in a small percentage of individuals are highly penetrant for ASD (i.e. copy number variants, single nucleotide variants) but also affect a diverse set of biological pathways [12–14]. Thus, the genomic landscape of risk mechanisms is highly diverse. Environmental factors as well as the interplay between genetic and environmental risk mechanisms are also likely important, though the magnitude of impact is still largely unknown [15].

Heterogeneity within ASD is a challenge for basic science attempts to understand the pathophysiological and neurodevelopmental mechanisms that lead to the disorder and for the development of effective psychopharmacological or behavioural treatments [16]. Decomposing heterogeneity across individuals and at multiple levels of analysis requires ‘big data’ approaches that are both ‘broad’ (i.e. large numbers of people) and ‘deep’, i.e. multiple levels of analysis within an individual—genetic and cellular architecture, brain structure and function, cognitive, behavioural, and clinical variation, assessing individuals across development, etc. [17].

### Variation of the ASD phenotype by sex, age and intellectual ability

ASD is at least three times more prevalent in males than females, and biological sex may be an important source of heterogeneity in ASD presentation. Lai and colleagues [18] recently summarised research on sex differences in ASD, covering potential mechanisms underlying the sex differential liability to possible sex differences in brain structure and function. Other factors may also affect the recognition and presentation of ASD symptoms in males and females, including potentially different patterns or profiles of symptoms and ‘compensatory’ or ‘masking’ of symptoms in females [18]. In addition, there is evidence from population studies that girls with similar levels of symptoms to boys are less likely to be diagnosed by community services [19], unless there are more substantial behavioural or cognitive difficulties [20]. In terms of clinical profile and behaviour, findings have been inconsistent. While a meta-analysis suggested lower levels of repetitive and restricted behaviours and interests (RRB) in females but comparable levels of social communication difficulties in males and females [19, 21], other studies have reported greater social communication difficulties and lower cognitive ability and adaptive function in females [22, 23]. Similarly, some studies have reported higher levels of anxiety in girls than boys with ASD and more externalising symptoms in boys [24–26]—but other studies have not [7]. Comparisons across studies are compromised by differences between samples such as varying rates of intellectual disability.

Age is another potential source of heterogeneity in individuals with ASD. There are some reports of reductions in ASD symptoms over early childhood [27] but also high variability in the trajectory over childhood and into early adolescence with some children showing stable high or low severity across development, while a minority significantly improve or worsen, respectively [28–33]. Several longitudinal studies have reported a reduction in ASD symptoms in adulthood, although functional outcomes for many individuals remain poor [34–36]. A number of longitudinal studies have reported lower levels of psychiatric symptoms in adolescence than in childhood [37, 38], and others have reported further reductions into adulthood [39] and even throughout the adult life course [40].

Variation in intellectual ability is included in DSM-5 as a ‘clinical specifier’, indicating its importance in driving heterogeneity of ASD. In many samples, lower IQ has been modestly but significantly associated with higher levels of ASD symptom severity [41, 42]. In contrast to the moderate association found in the general population between low IQ and increased levels of externalising disorders [43, 44], some studies have reported that in population-derived samples, this association was only present in adolescents (and not children) with ASD [7, 38]. A meta-analysis focusing on anxiety disorders in ASD revealed complex associations with IQ, finding that social anxiety was more common in studies with lower IQ samples but that obsessive-compulsive disorder and separation anxiety were higher in studies with higher IQ samples [45].

### Clinical characterisation of the EU-AIMS LEAP cohort

As described in the companion paper [46], as part of the EU-AIMS clinical research programme [47–49], we established the Longitudinal European Autism Project (LEAP). Here, we report on the baseline clinical assessment of the EU-AIMS LEAP cohort. The paper will first describe the cohort and its clinical characteristics. Then, taking advantage of the size and heterogeneity of the cohort, we will examine whether there are sex, age and IQ differences on measures of core ASD symptoms and levels of commonly co-occurring psychiatric symptoms.

## Methods

### Participants

In this multi-site study, participants were recruited between January 2014 and March 2017 across six European specialist ASD centres: Institute of Psychiatry, Psychology and Neuroscience, King’s College London (IoPPN/KCL, UK), Autism Research Centre, University of Cambridge (UCAM, UK), University Medical Centre Utrecht (UMCU, Netherlands), Radboud University Nijmegen Medical Centre (RUNMC, Netherlands), Central Institute of Mental Health (CIMH, Germany) and the University Campus Bio-Medico (UCBM) in Rome, Italy (see Table 1 for recruitment information by site). In addition, twins discordant for ASD were recruited at Karolinska Institutet, Sweden—however, twins were not included in the case-control comparisons reported below. Participants were recruited from a variety of sources including existing volunteer databases, existing research cohorts, clinical referrals from local outpatient centres, special needs schools, mainstream schools and local communities. Based on parent- or self-reported ethnicity, most participants were Caucasian white (73%). The remaining participants were described as either of mixed race (6%), Asian (2%), black (1%) or other (2%). For 16% of participants information on ethnicity was either not provided (12%) or missing (4%). Annual household income was measured on an 8-point-scale ranging from <£25,000 to >£150,000, with the median annual household income being estimated at £30,000–£39,999. Highest household parental education was coded on a 5-point scale ranging from primary education to postgraduate qualifications; 61% of households had at least one parent with education beyond a high school diploma (i.e. with an undergraduate degree from university). At each site, an independent ethics committee approved the study. All participants (where appropriate) and their parent/legal guardian provided written informed consent.

**Table 1:** Number of participants recruited by each site according to schedule and diagnostic group

	Total		Adults		Adolescents		Children		Mild ID	
	ASD	TD/ID	ASD	TD	ASD	TD	ASD	TD	ASD	ID
London (KCL)	159	89	55	38	41	19	32	14	31	18
Cambridge (UCAM)	59	34	17	14	22	10	17	10	3	0
Mannheim (CIMH)	36	38	7	5	20	25	7	8	2	0
Nijmegen (RUNMC)	117	74	24	13	31	28	32	22	30	11
Rome (UCBM)	22	19	21	19	0	0	0	0	1	0
Utrecht (UMCU)	44	46	18	20	12	12	13	14	1	0
Total	437	300	142	109	126	94	101	68	68	29

*ASD* autism spectrum disorder, *TD* typically developing, *Mild ID* intellectual disability

### Inclusion/exclusion criteria

Participant inclusion criteria for the ASD sample were an existing clinical diagnosis of ASD according to DSM-IV [50], DSM-IV-TR [51], DSM-5 [5] or ICD-10 [52] criteria and age between 6 and 30 years. ASD diagnoses were based on a comprehensive assessment of the participant’s clinical history and/or current symptom profile, depending on when the participant was originally identified at that site. In addition, we assessed ASD symptoms using the Autism Diagnostic Observation Schedule (ADOS; [53, 54]) and the Autism Diagnostic Interview-Revised (ADI-R; [55]). However, individuals with a clinical ASD diagnosis who did not reach cut-offs on these instruments were not excluded. Clinical judgement has been found to be more stable than scores on individual diagnostic instruments alone [56], reflecting the moderate-to-good but still imperfect accuracy of such tools [57].

Exclusion criteria included significant hearing or visual impairments not corrected by glasses or hearing aids, a history of alcohol and/or substance abuse or dependence in the past year and the presence of any MRI contraindications (e.g. metal implants, braces, claustrophobia) or failure to give informed written consent to MRI scanning (or to provide contact details for a primary care physician at centres where this is a pre-condition for scanning). Participants were purposively sampled to enable in depth experimental characterisation of potential biomarkers (including MRI scans). Therefore, we excluded individuals with low IQ (<50) as core measures (e.g. most cognitive tasks and MRI scanning without sedation) were deemed difficult to administer in this group. Participants who did not complete an IQ assessment were excluded (controls: *n* = 7, ASD: *n* = 10). In the TD group, individuals who had a *T* score of 70 or higher on the self-report (1 adult) or parent-report form (1 adolescent, 3 children) of the Social Responsiveness Scale [58] were also excluded.

In the ASD sample, psychiatric conditions (except for psychosis or bipolar disorder) were allowed as up to 70% of people with ASD have one or more psychiatric disorders [7] and reflect DSM-5 that allows co-occurring psychiatric disorders alongside an ASD diagnosis [5]. In future individual biomarker analyses, additional exclusion criteria or sub-grouping may then be applied (e.g. ADI-R cut-offs, medication-free, etc.).

Exclusion criteria of the TD/ID group were the same as described above for the ASD participants with the exception that in the TD group parent- or (where appropriate) self-report of a psychiatric disorder was also an exclusion criteria.

### Study schedules

Participants were split into four study schedules depending on their age and cognitive ability level. Three schedules included individuals with IQ in the typical range (≥75) (*children*: aged 6–11 years, *adolescents*: aged 12–17 years and *adults*: aged 18–30 years). At two sites (KCL, RUNMC)^1^, adolescents and adults (aged 12–30 years) with ASD and mild intellectual disabilities (mild ID; defined by IQ between 50 and 74^2^) were also recruited alongside age- and IQ-matched individuals without ASD (*mild ID* group). Each schedule received a tailored and largely comparable study protocol to take into account differences in age and cognitive level [46]. Within each age band (children, adolescents, adults), participants were recruited with a similar male:female ratio (3:1) and IQ composition so that predicted cognitive/biological differences can be compared across sex and developmental stages. Likelihood ratio tests confirmed that the targeted male:female ratio did not differ significantly across schedules (*x* ^*2*^(2) = 1.41, *p* = .494) and study sites (*x* ^*2*^(5) = 2.69, *p* = .754), as well as between ASD and TD groups within each age band (all *p* > .1).

### Clinical measures—ASD symptomatology

Given the cautious conclusions of recent reviews of ASD symptom measures as potential endpoints for clinical trials [59–61], we used a range of different measures of ASD symptoms (a full list of all clinical measures is reported in the Additional file 1: Table 3). These various ASD symptom measures have complementary strengths and limitations, relevant to our clinical and conceptual understanding of measurement of ASD symptomatology [57]. The parent-report ADI-R algorithm gives historical/early developmental symptom severity; the ADOS is an observational measure of current symptom severity. Both are diagnostic instruments. The ADOS has a standardised ‘calibrated severity score’, that is equivalent across different modules while the ADI-R produces raw algorithm scores in the three core ASD behavioural domains but is more susceptible to skew. The ADI and ADOS were not administered to the typically developing controls or mild ID cases without ASD. In addition, dimensional measures of ASD symptomatology were derived from a variety of questionnaires (described below). Each of these questionnaires was parent rated and/or self rated depending on age and cognitive level (see Table 2 for a summary of parent-report and participant self-report questionnaires). The use of both parent and self-report in a subsample will allow us to determine if the pattern of age and sex differences in ASD and associated psychiatric symptoms varies by respondent, which will have implications both for mapping putative biomarkers onto the ASD phenotype and for their use as outcomes in clinical trials. The *Social Responsiveness Scale, Second Edition* (SRS-2; [58]) is a parent-reported symptom questionnaire suitable across the whole age range (and is sex normed) that in addition has a self-report companion measure suitable for adolescents and adults. Other questionnaire measures (*Autism Spectrum Quotient* (AQ; [62–64]); *Children’s Social Behaviour Questionnaire* (CSBQ; [65])/*Adult Social Behaviour Questionnaire* (ASBQ; [66]) are designed as more dimensional/trait measures of ASD severity and have different versions across the age span. The inclusion of multiple dimensional measures of ASD symptom severity will allow us to test which measure best relates to neurobiological or neurocognitive biomarkers and is most sensitive to change over time. Other questionnaires measure aspects of the ASD phenotype not well captured by the SRS-2, including atypical sensory responses (*Short Sensory Profile* (*SSP*; [67]) and repetitive, rigid and stereotyped behaviours (*Repetitive Behavior Scale-Revised* (*RBS-R*; [68]).

**Table 2:** Summary of parent-report and participant self-report questionnaires

Phenotypic measures	Adults (TD)	Adults (ASD)	Adolescents (TD/ASD)	Children (TD/ASD)	Mild ID (ID/ASD)
Dimensional measures of ASD symptoms					
Social Responsiveness Scale-2nd Edition (SRS-2),	S	S & P	S & P	P	P
Children’s Social Behaviour Questionnaire (CSBQ)	-	-	P	P	P
Adults’ Social Behaviour Questionnaire (ASBQ)	S	S & P	-	-	P (>18 years)
Autism Spectrum Quotient (AQ)—adult version	S	S & P	-	-	-
Autism Spectrum Quotient (AQ)—adolescent version	-	-	P	-	-
Autism Spectrum Quotient (AQ)—child version	-	-	-	P	P
Repetitive Behaviour Scale-Revised (RBS-R)	-	P	P	P	P
Short Sensory Profile (SSP)	-	P	P	P	P
Psychiatric symptoms					
DSM-5 ADHD rating scale	S	S & P	P	P	P
Beck Anxiety Inventory	S	S	S	P	P
Beck Depression Inventory	S	S	S	P	P

*S* self-report (completed by participant), *P* parent-report (completed by primary carer or parent of participant), *S & P* self- and parent-report administered; *-* not administered

The *Autism Diagnostic Observation Schedule (ADOS*; [53, 54]), a standardised social interaction observation assessment, was used to assess current symptoms in ASD participants (module 2 for 2 participants, module 3 for 154 participants, module 4 for 208 participants). Calibrated Severity Scores (CSS) for Social Affect (SA), Restricted and Repetitive Behaviours (RRB) and Overall Total were computed [69, 70], which provide standardised autism severity measures that account for differences in the modules administered. The *Autism Diagnostic Interview-Revised* (*ADI-R*; [55]), a structured parent interview, was completed with parents/carers of ASD participants. Standard algorithm scores which combine current and historical symptom information were computed for Reciprocal Social Interaction (Social), Communication, and Restricted, Repetitive and Stereotyped Behaviours and Interests (RRB). Current ADI-R scores were available on a subset of the ASD sample (356/414 (86%)) but are not reported in the current paper. Where ADOS and ADI-R scores from previous assessments were available (ADOS: within the past 12 months for children/past 18 months for all other schedules; ADI-R: at any historical point since we report the 4 to 5 years/ever algorithm scores), these assessments were not repeated.

The *Social Responsiveness Scale, Second Edition* (*SRS-2*; [58]) is a quantitative measure comprising 65 items asking about characteristic autistic behaviour over the previous 6 months. Each item is scored using a ‘0’ (not true) to ‘3’ (almost always true) on a Likert scale. The total raw score is transformed into sex-specific *T* scores, and here, we report both raw and sex-standardised scores. Parent report was used for all participants with ASD and mild ID, as well as children and adolescents with typical development. Adults with ASD additionally completed the self-report form. Adults with typical development only completed the self-report form as, for feasibility reasons, in this schedule, parents were not enrolled in the study.

The *Repetitive Behavior Scale-Revised* (*RBS-R*; [68]) assesses restricted repetitive behaviours associated with ASD. Parents or caregivers rate 43 behaviours (e.g. ‘arranges certain objects in a particular pattern or place’; ‘need for things to be even or symmetrical’) on a scale of 0–3, where 0 indicates the behaviour does not occur and 3 indicates the behaviour does occur and is a severe problem.

Sensory processing atypicalities were measured using the SSP [67]. This parent-report questionnaire comprises 37 items, where each item is scored on a 5-point Likert-rating scale from 1 (always occurs) to 5 (never occurs). The SSP is based on the sensory profile [71]. Lower scores on the SSP are indicative of greater impairment.

The CSBQ [65] is a 49-item parent-report questionnaire that is specifically useful in assessing behaviour atypicalities across the entire ASD spectrum. Adults received the ASBQ for either self or parent report, composed of 44 items [66].

The AQ [62–64]) is a continuous self- or parent-report measure that quantifies the degree to which children, adolescents or adults of average intelligence show behavioural characteristics associated with ASD. The AQ consists of 50 statements asking about habits and personal preferences. Each statement is rated by the participant or parent/carer on a 4-point Likert-rating scale from ‘definitely agree’, ‘slightly agree’, ‘slightly disagree’ to ‘definitely disagree’. While adult participants completed the AQ by self-report, the adolescent version is parent report but is otherwise composed of the same items compared to the adult AQ. The AQ-Child also entails parent-report, yet items that were not age appropriate in the adolescent/adult questionnaire were revised accordingly.

### Intellectual ability

Level of intellectual abilities was assessed using the Wechsler Abbreviated Scales of Intelligence—Second Edition, WASI-II [72] or—in countries where the WASI is not translated (i.e. The Netherlands, Germany and Italy)—the four-subtest short forms of the German, Dutch or Italian WISC-III/IV [73, 74] for children or WAIS-III/IV [75, 76] for adults. The shortened versions were used for feasibility reasons to not further prolong the testing sessions for participants. All versions included two verbal subscales (vocabulary, similarities) and two non-verbal subscales (block design, matrix reasoning). To standardise data across sites, IQ was prorated from two verbal subtests (vocabulary and similarities) and two performance subtests (matrix reasoning and block design) using an algorithm developed by [77] that produces an estimated IQ score that is highly correlated (*r* = .93) with a full-Scale IQ obtained by administering the complete test. Age-appropriate national population norms were available for each participating site, and these were used to derive standardised estimates of an individual’s intellectual functioning. Where recent IQ scores from previous assessments were available (less than 12 months in children; less than 18 months in adolescents and adults), IQ tests were not repeated.

### Clinical measures—co-occurring psychiatric symptoms

The *Beck Depression Inventory—Second Edition* (BDI-II; [78]) is a 21-item inventory measuring the severity of characteristic attitudes and symptoms associated with depression. Each item contains four possible responses, which range in severity from 0 (e.g. ‘*I do not feel sad’*) to 3 (e.g. ‘*I am so sad or unhappy that I can’t stand it’*). Participants are asked to provide answers based on the way they have been feeling over the past month, including the assessment day. The self-report version of the BDI-II was administered to adult participants. Parents/caregivers completed the depression subscale of the *Beck Youth Inventories* (BYI-II; [79]) for children and adolescents/adults with mild ID. Adolescents were given the depression subscale of the BYI-II as self-report.

The *Beck Anxiety Inventory* (BAI; [80]) is a well-validated 21-item inventory probing for common symptoms of anxiety. Participants rate each item along different levels of symptom severity experienced over the past month from 0 = not at all to 3 = severely. The self-report version of the BAI was administered to adult participants. Children and adolescents/adults with mild ID were given the anxiety subscale of the Beck Youth Inventories (BYI-II; [79]) as parent-report, while adolescents completed the anxiety subscale of the BYI-II as self-report.

The *DSM-5 rating scale of attention-deficit/hyperactivity disorder (ADHD)* covers 18 items measuring the presence of inattention and hyperactive/impulsive symptoms in the past 6 months, each evaluated on a 0–3 scale (0 = not at all to 3 = very often). In children, six or more responses scored with 2 (often) or 3 (very often) to either (or both) the inattention and hyperactivity/impulsivity domains indicate clinical concern. Depending on age and ability level, either parent- or self-report forms were administered.

### Quality control procedures

Appropriate to a multi-centre, cross-national study, we established quality control procedures around training, data collection and data entry and checking. We had cross-site training sessions for collecting clinical data, the ADOS and ADI-R were administered and scored by qualified/certified personnel and the study was regularly monitored according to good clinical practice standards. Of the total number of ADI-R assessments (4–5 ever/diagnostic) administered to participants (*N* = 414), *N* = 162 were re-used from previous studies, while for the ADOS (*N* = 364), a total of *N* = 61 were re-used (all completed within the previous 12 months). Prior to data analysis, a series of quality control procedures were adopted to maximise coherence and comparability of data. This involved initial randomised double data entry of 10% of cases at each site for core clinical measures (e.g. ADI-R, ADOS, IQ data). If a significant level of incorrect/inconsistent data was identified, all data was checked against the original paper forms. Other procedures also included impossible values/range checks of all items, sub-scales and total scores for interview and questionnaire measures, duplicated entry detection and correction, as well as data audits and checks of scoring algorithms. When missing data was present, site coordinators were asked to secure the information if possible.

Across all clinical measures, we have applied a prorating approach to deal with missing scores. Prorating replaces the missing score for a given participant with her/his mean score on other items on the same sub-scale. Prorating was only applied if less than 20% of scores on the same sub-scale were missing. For a higher percentage of missing scores, prorating was not applied (i.e. data for these participants was recorded as missing).

### Statistical analysis

Statistical analysis was performed with the following objectives:

1. To examine whether there are age and/or sex differences in the severity of ASD symptoms by comparing individuals with ASD across different age groups (children, adolescents, adults);
2. To examine whether differences in age (i.e. ADOS) or sex (i.e. ADI-R, ADOS) are observed on diagnostic instruments as well as on continuous measures of ASD symptomatology (i.e. SRS-2, CSBQ/ASBQ, AQ, RBS-R, SSP) and whether these patterns are similar or different across parent- and self-report measures;
3. To characterise the association between ASD symptoms and level of intellectual functioning;
4. To characterise the severity of co-occurring psychiatric symptoms (i.e. ADHD, anxiety, depression) in individuals with ASD and to examine how these relate to age, sex and IQ.

Linear mixed-effects models were fit using a maximum likelihood estimation method and were executed using STATA software 14.0 [81]. Differences in ASD symptomatology between individuals with ASD relating to age, sex and IQ were analysed by restricting the analysis to participants with ASD only since by definition ASD participants will score more highly than controls on ASD symptom measures. Each model (except for ADI-R diagnostic scores) included fixed main effects for study schedules (children, adolescents, adults and mild ID) and sex (male, female), as well as their interaction. In this paper, we treat age and IQ in two ways. First, both for clinical ‘face validity’ and to allow the comparison between the clinical characteristics of the LEAP cohort to previously published samples—often comprised of children, adolescents or adults only, with or without intellectual disability and not with the heterogeneity present in our cohort by design—we analyse and present the clinical data in the main paper according to the age/IQ-defined schedules outlined above. Second, in the (Additional file 2: Table S1), we present scores on some of the key measures continuously by age and IQ as this maximises the power of the large sample and recognises the arbitrary nature of creating age and IQ ‘groups’ by ‘binning’ the sample into pre-defined age and IQ subgroups. For the analysis by schedule, significant main and interaction effects were further explored using post-estimation methods including contrasts (Bonferroni-corrected for the number of post hoc comparisons for each measure separately) and margin plots. Log-transformed variables were used where appropriate to meet normality assumptions (RBS-R, SSP). A random effect for site was included in all models to take into consideration the multi-level nature of the data, as well as to account for site heterogeneity across outcome measures. Intraclass correlation coefficients (ICCs) reflecting the ratio of between-site variance to total variance are reported (see Table 4). All models included a continuous measure of IQ (full-scale IQ) as a covariate (Additional file 3: Table S2). Linear mixed models report chi-square coefficients and *p* value. Effect sizes were calculated following [82] by dividing the difference in marginal means by the square root of the variance at the within-participant level. This measure of effect size is equivalent to Cohen’s *d* or standardised difference [83], where an effect size of 0.2 to 0.3 is taken to be a small effect, 0.5 a medium effect and greater than 0.8 a large effect. For the analyses reported in the (Additional file 2: Table S1) that treat age and IQ as continuous variables, we performed linear mixed-effects models to take into account site effects yet replacing the categorical age/ability level variable with continuous measures of chronological age and IQ.

## Results

Participant characteristics are shown in Table 3.

**Table 3:** Sample characteristics

		Total		Adults		Adolescents		Children		Mild ID	
		ASD	TD/ID	ASD	TD	ASD	TD	ASD	TD	ASD	ID
Sex	N	437	300	142	109	126	94	101	68	68	29
	Males (%)	72.3	65	72.5	67	77	69.1	71.3	61.8	64.7	51.7
	Females (%)	27.7	35	27.5	33	23	30.9	28.7	38.2	35.3	48.3
Age (in years)	M	16.68	17.22	22.79	23.10	14.86	15.33	9.40	9.52	18.09	19.30
	SD	5.80	5.94	3.37	3.27	1.73	1.73	1.58	1.54	4.27	4.97
	Range	6.08–30.60	6.24 -30.78	18.02–30.60	18.07–30.78	12.07–17.90	12.04–17.99	6.08–11.97	6.24–11.98	11.50–30.19	12.92–30.24
Full-scale IQ	M	97.61	104.57	103.99	109.15	101.59	106.58	105.29	111.46	65.84	63.39
	SD	19.74	18.26	14.82	12.60	15.68	13.18	14.76	12.69	7.70	8.00
	Range	40^a^–148	50–142	76–148	76–142	75–143	77–140	74–148	76–142	40^a^–74	50–74

*ASD* autism spectrum disorder, *TD* typically developing, *Mild ID* intellectual disability

^a^There are 3 individuals with a full-scale IQ <50

### Demographics

In the total sample, the mean (SD) chronological age was 16.9 (5.9) years, with similar distributions of age for individuals with ASD (*M* = 16.7, *SD* = 5.8) and TD/mild ID individuals (*M* = 17.2, *SD* = 5.9), *x* ^*2*^(1) = 1.84, *p* = .175. Of the 737 participants, 511 were men and 226 were woman (2.3:1 male-female ratio). While overall, the male-female ratio was significantly but only slightly higher across individuals with ASD (2.6:1) relative to TD/mild ID individuals (1:9:1) (*x* ^*2*^(1) = 5.49, *p* = .019), it was not significant within each age band (all *p* > .1). For annual household income, there was a significant interaction between diagnosis and schedule (*x* ^*2*^(4) = 26.10, *p* = .0001), with individual comparisons indicating that household income was significantly higher in TD children compared to children with ASD (*x* ^*2*^(1) = 13.61, *p* = .0009). For both paternal (*x* ^*2*^(4) = 10.86, *p* = .028) and maternal education (*x* ^*2*^(4) = 19.08, *p* = .0008), a significant interaction between diagnosis and schedule was found. Individual contrasts revealed that the level of paternal and maternal education was significantly higher in TD children relative to children with ASD (*x* ^*2*^(1) = 5.11, *p* = .024 and *x* ^*2*^(1) = 6.55, *p* = .042 respectively). There were no differences in ethnicity between TD/mild ID and ASD participants overall and within each age band (all *p* > .4).

### Site effects

The random effect for site included in all the models was significant for all the key demographic and diagnostic measures except for sex and ADOS Total and Social Affect CSS (see Table 4). The ICCs shown in Table 4 indicate that while the effect of site was large for age (~25%), reflecting the variable recruitment targets across age schedules and across sites (see Table 1), for other measures, it was low to moderate, being less than 1% for sex ratio, less than 6% for IQ, between 9 and 15% for ADI-R scores and less than 8% for ADOS scores.

**Table 4:** Summary of variation between sites in demographic and behavioural characteristics and level of ASD symptomatology for individuals with ASD only

	Ranges across sites				Variance				
	Minimum	Maximum	Mean	SD	Overall mean (SD)	Within sites	Between sites	ICC^a^	*x* ^2^ sig. value
Chronological age [years:months]	6:07–19:8	24:5–30:6	14:8–25:0	3:2–6:3	16:7 (5:8)	29.87	9.91	.249	*p* < .0001
Sex, % of male participants			66.1–80.6		72.3 (4.48)	0.46	<.01	<.001^b^	n.s.^c^
Verbal IQ	45^d^–70	130–160	93–110	14–21	97 (19)	382.18	12.61	.031	*p* < .0001
Nonverbal IQ	45^d^–68	134–150	93–107	16–23	98 (21)	430.82	24.39	.054	*p* = .0001
Full-scale IQ	40^d^–73	128–148	96–105	12–22	98 (20)	373.45	16.35	.042	*p* = .001
ADI-R									
Social interaction	0–4	24–29	12–19	6–7	17 (7)	42.26	4.33	.093	*p* < .0001
Communication	0–3	17–26	9–16	5–5	13 (6)	28.10	4.97	.150	*p* < .0001
RRB	0–1	8–12	3–5	2–4	4 (3)	6.06	.85	.122	*p* < .0001
ADOS—CSS									
Total	1	10^c^	5–9	2–3	5 (3)	2.77	.35	<.001^b^	n.s.
SA	1	10^c^	6–7	2–3	6 (3)	6.88	.11	.016	n.s.
RRB	1	9–10	4–8	2–3	5 (3)	7.29	.60	.076	*p* < .0001
Sample size^e^	22	159	72	54					

*ICC* intraclass correlation coefficient, *ADI*-R Autism Diagnostic Interview-Revised, *ADOS CSS Total, SA, RRB* Autism Diagnostic Observation Schedule Calibrated Severity Scores for Total, Social Affect and Restricted and Repetitive Behaviours, *IQ* intelligence quotient, n.s. not significant

^a^The ratio of between-site variance to total variance

^b^ICC truncated at zero

^c^The highest possible score (i.e. ceiling) on the instrument

^d^There are 3 individuals with a full-scale IQ <50 (All ASD)

^e^Sample size variation of individuals with ASD across sites (minimum/maximum, mean and standard deviation of number of participants with ASD recruited at sites)

### Diagnostic ASD measures—sex and age effects

**Fig. 1:**
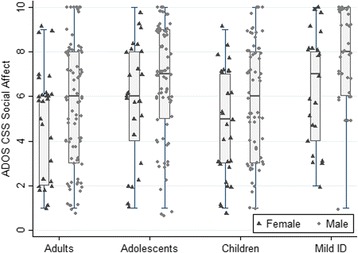
Boxplot of ADOS CSS Total scores by sex and for each schedule (ASD participants only)

On the ADOS, male ASD participants had significantly higher CSS Total (*x* ^*2*^(1) = 15.81, *p* = .0001, *d* = .46) and CSS Social Affect (SA) (*x* ^*2*^(1) = 12.71, *p* = .0004, *d* = .44) than females with ASD and was approaching significance for CSS Restricted and Repetitive Behaviours (RRB) (log-transformed, *x* ^*2*^(1) = 3.15, *p* = .076, *d* = .22) (see Table 5 and Fig. 1). A significant interaction between sex and schedule was found for CSS Total (*x* ^*2*^(4) = 16.97, *p* = .002) and CSS SA (*x* ^*2*^(4) = 13.32, *p* = .009). Individual comparisons indicated that only in adolescents, males had significantly higher ADOS CSS Total than females (*x* ^*2*^(1) = 5.93, *p* = .04, *d* = .56).

**Table 5:** Sex differences for key measures for ASD and TD/ID participants (pooled across schedules)

	ASD		TD/ID	
	Males	Females	Males	Females
Autism symptomatology measures				
*ADI—Social*	17.01 (6.78)	15.36 (6.89)	–	–
*ADI—Communication*	13.55 (5.86)	12.58 (5.33)	–	–
*ADI—RRB*	4.57 (2.66)	3.74 (2.52)	–	–
*ADOS—CSS Total*	5.73 (2.83)	4.60 (2.49)	–	–
*ADOS—CSS SA*	6.31 (2.66)	5.46 (2.56)	–	–
*ADOS—CSS RRB*	5.03 (2.86)	4.30 (2.69)	–	–
*SRS-2* ^*a*^	71.50 (11.70)	73.65 (12.18)	47.49 (9.97)	48.07 (9.17)
*SRS-2* ^*b*^	62.37 (9.91)	66.48 (11.14)	48.48 (6.08)	46.55 (6.13)
*CSBQ* ^*a*^	46.86 (17.01)	46.94 (15.62)	7.55 (12.56)	6.30 (8.50)
*ASBQ* ^*a*^	32.78 (16.76)	32.61 (16.55)	14.67 (15.17)	22.11 (20.76)
*ASBQ* ^*b*^	30.34 (15.08)	37.37 (15.75)	8.11 (8.49)	7.53 (8.97)
*AQ—child*	94.26 (18.00)	92.76 (17.39)	45.21 (17.95)	29.70 (10.07)
*AQ—adolescents*	95.78 (17.66)	96.32 (18.02)	48.92 (20.43)	44.75 (20.67)
*AQ—adults*	81.03 (18.86)	88.06 (20.78)	49.46 (14.88)	43.10 (14.05)
*RBS-R* ^*a*^	17.16 (14.01)	15.76 (13.48)	2.58 (9.43)	2.42 (5.02)
*SSP* ^*a*^	138.12 (27.78)	138.15 (26.83)	175.17 (17.00)	175.75 (17.46)
Psychiatric symptom measures				
*ADHD—inattentiveness* ^*a*^	4.75 (3.13)	4.05 (3.18)	1.34 (2.19)	1.23 (2.58)
*ADHD—hyperactivity/impulsivity* ^*a*^	2.98 (2.91)	2.47 (2.71)	0.57 (1.57)	0.54 (1.63)
*Anxiety* ^*a*^	48.52 (8.68)	49.14 (9.91)	40.27 (7.75)	38.64 (6.00)
*Depression* ^*a*^	51.42 (11.82)	50.44 (8.09)	41.76 (10.47)	39.77 (4.97)

*ADI* Autism Diagnostic Interview–Revised, *ADOS CSS Total, SA, RRB* Autism Diagnostic Observation Schedule Calibrated Severity Scores for Total, Social Affect and restricted and repetitive behaviours; *SRS-2* Social Responsiveness Scale–2, *CSBQ, ASBQ* Children’s Social Behaviour Questionnaire (parent-report, administered to children, adolescents), Adults’ Social Behaviour Questionnaire (parent-report, administered to adults) scores cannot be pooled across age groups, *RBS-R* Repetitive Behavior Scale–Revised, *SSP* Short Sensory Profile, *AQ* Autism Spectrum Quotient (children, adolescents and adult version; scores cannot be pooled across age group

^a^Parent-report

^b^Self-report

A similar pattern of results was also observed on the ADI-R, where male ASD participants had more severe scores than female ASD participants on the Social (*x* ^*2*^(1) = 5.98, *p* = .015, *d* = .27), and Restricted and Repetitive Behaviours (RRB) domain (*x* ^*2*^(1) = 7.81, *p* = .005, *d* = .30) but not Communication domain (*x* ^*2*^(1) = 2.27, *p* = .131. *d* = .19). No significant effect of schedule was observed for ADI-R and ADOS scores (see Table 6).

**Table 6:** ADI-R and ADOS scores by schedule for individuals with ASD only

	Total	Adults	Adolescents	Children	Mild ID
*ADI-R—Social*	16.54 (6.85) *n* = 411	15.31 (6.87) *n* = 132	17.27 (6.55) *n* = 123	15.38 (6.76) *n* = 94	19.45 (6.57) *n* = 62
*ADI-R—Communication*	13.13 (5.72) *n* = 414	12.19 (5.76) *n* = 132	13.63 (5.63) *n* = 123	13.29 (5.75) *n* = 96	13.87 (5.62) *n* = 63
*ADI-R—RRB*	4.33 (2.65) *n* = 414	4.23 (2.62) *n* = 132	4.28 (2.71) *n* = 123	4.68 (2.79) *n* = 96	4.14 (2.36) *n* = 63
*ADOS—CSS Total*	5.39 (2.78) *n* = 362	4.84 (2.80) *n* = 110	5.78 (2.77) *n* = 102	4.98 (2.65) *n* = 91	6.39 (2.65) *n* = 59
*ADOS—CSS SA*	6.06 (2.65) *n* = 362	5.49 (2.70) *n* = 110	6.44 (2.55) *n* = 102	5.55 (2.58) *n* = 91	7.25 (2.38) *n* = 59
*ADOS—CSS RRB*	4.81 (2.83) *n* = 362	4.80 (2.76) *n* = 110	4.84 (2.61) *n* = 102	4.84 (3.07) *n* = 91	4.75 (3.00) *n* = 59

*ASD* (autism spectrum disorder), Mild ID (intellectual disability), *ADI-R* Autism Diagnostic Interview–Revised, *ADOS* Autism Diagnostic Observation Schedule

### Dimensional ASD measures—sex and age effects

**Fig. 2:**
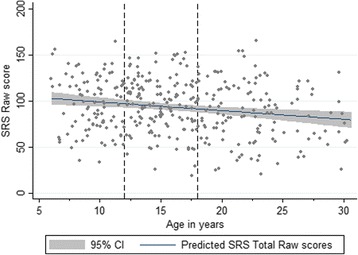
SRS-2 raw scores (parent-report) by chronological age (ASD participants only)

**Fig. 3:**
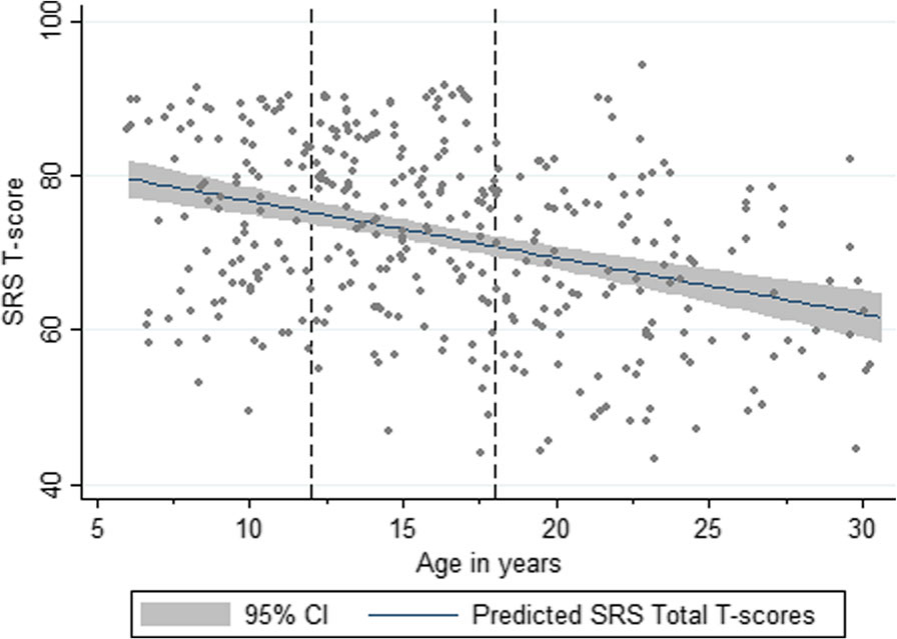
SRS-2 Total scores (parent-report) by chronological age (ASD participants only)

Parent-report and self-report data were analysed separately. For parent-reported SRS-2 raw scores, no significant sex differences were observed within the ASD group (*x* ^*2*^(1) = 0.01, *p* = .939). There were however significant differences in SRS-2 raw scores across the various schedules (*x* ^*2*^(3) = 16.82, *p* = .0008). Follow-up contrasts (Bonferroni-corrected *p* values) indicated that adults had significantly lower SRS-2 raw scores compared to children (*x* ^*2*^(1) = 13.93, *p* = .0006, *d* = .62) and adolescents (*x* ^*2*^(1) = 10.34, *p* = .0039, *d* = .52) (see Fig. 2) but not compared to adolescents/adults with ASD and mild ID (*x* ^*2*^(1) = 4.82, *p* = .084, *d* = .49). For parent-reported SRS-2 *T* scores (age- and sex-adjusted), while there were no significant sex differences within the ASD group (*x* ^*2*^(1) = 2.58, *p* = .108), SRS-2 *T* scores differed significantly across the various schedules (*x* ^*2*^(3) = 65.70, *p* < .0001). Follow-up contrasts indicated that adults had significantly lower SRS-2 *T* scores compared to children (*x* ^*2*^(1) = 51.16, *p* < .0001, *d* = 1.19) and adolescents (*x* ^*2*^(1) = 46.52, *p* < .0001, *d* = 1.10), as well as compared to adolescents/adults with ASD and mild ID (*x* ^*2*^(1) = 12.43, *p* = .001, *d* = .80) (see Fig. 3). The interaction between sex and schedule was not significant (*x* ^*2*^(3) = 6.43, *p* = .169).

Adolescents and adults also completed the SRS-2 as self-report. On this measure, females had significantly higher SRS-2 raw scores (*x* ^*2*^(1) = 6.81, *p* = .009, *d* = .49) and *T* scores (*x* ^*2*^(1) = 7.02, *p* = .008, *d* = .50) than males overall. A significant interaction between schedule and sex was also observed for SRS-2 raw scores (*x* ^*2*^(1) = 9.60, *p* = .008) and SRS-2 *T* scores (*x* ^*2*^(1) = 9.89, *p* = .007). Follow-up tests revealed that adult ASD females reported significantly higher SRS-2 raw scores (*x* ^*2*^(1) = 8.38, *p* = .008, *d* = .60) and *T* scores (*x* ^*2*^(1) = 8.63, *p* = .007, *d* = .60) than adult ASD males, but there were no sex differences in adolescents.

In contrast to parent-reported SRS-2 *T* scores, adults had significantly higher self-reported SRS-2 *T* scores (*x* ^*2*^(1) = 6.57, *p* = .010, *d* = .36) and SRS-2 raw scores (*x* ^*2*^(1) = 6.55, *p* = .011, *d* = .36) than adolescents. On both the parent-report versions of the CSBQ and ASBQ, which were analysed separately due to differences in item and sub-scale structure, no main effect of sex or schedule and no significant sex by schedule interaction were observed. In contrast, for adults with ASD completing the ASBQ as self-report, females reported significantly higher scores than males (*x* ^*2*^(1) = 7.57, *p* = .006, *d* = .48).

Data on the AQ was analysed separately for children, adolescents and adults because different versions of the measure were used. On the Adult-AQ (self-report), sex differences were approaching significance with females having higher scores than males (*x* ^*2*^(1) = 3.40, *p* = .065, *d* = .39). Some group effects were found on the AQ-Adolescent, where adolescents with ASD and ID had significantly higher AQ scores than adolescents with ASD without ID (*x* ^*2*^(1) = 7.69, *p* = .006, *d* = .93).

Prior to analysis, total scores of the RBS-R were log transformed to meet normality assumptions. There was no significant effect of sex (*x* ^*2*^(1) = .32, *p* = .569) but a significant main effect of schedule (*x* ^*2*^(3) = 27.13, *p* < .0001), with adults having significantly lower RBS-R scores relative to children (*x* ^*2*^(1) = 26.20, *p* < .0001, *d* = .91) and adolescents (*x* ^*2*^(1) = 11.98, *p* = .001, *d* = .57). There was no significant interaction effect between sex and schedule.

On the SSP (using log-transformed total scores), no main effect of sex or schedule and no significant sex by schedule interaction were observed.

### Intellectual functioning

The mixed-effects analysis revealed a significant interaction between schedule and diagnosis for full-scale IQ scores (*x* ^*2*^(4) = 25.13, *p* = .0001, see Table 3), with significantly higher IQ scores in TD individuals compared to participants with ASD in the adult (*x* ^*2*^(1) = 8.60, *p* = .01, *d* = .39), adolescent (*x* ^*2*^(1) = 7.79, *p* = .02, *d* = .38) and children age groups (*x* ^*2*^(1) = 8.23, *p* = .017, *d* = .37). No significant differences in intellectual functioning were found between individuals with/without ASD and mild ID.

Examining the association between measures of ASD symptomatology and IQ (full-scale IQ) in individuals with ASD only, there were significant, albeit weak negative correlations between ADOS Total CSS and IQ (*r* = −.23; *n* = 358; *p* < .0001), as well as between ADOS Social Affect CSS and IQ (*r* = −.23; *n* = 358; *p* < .0001), with higher IQs being associated with lower symptom levels. There was no significant association between ADOS RRB CSS and IQ. Scores on the ADI-R Social domain (*r* = −.22; *n* = 404; *p* < .0001) and ADI-R Communication domain (*r* = −.12; *n* = 407; *p* = .04), but not ADI-R RRB domain (*r* = .01; *n* = 407; *p* = .782), were also significantly associated with IQ. On dimensional measures of ASD symptom severity significant negative correlations between SRS-2 Total *T* scores (parent-report) and IQ (*r* = −.23; *n* = 350; *p* < .0001) see (Additional file 3: Table S2), between SRS-2 raw scores (parent-report) and IQ (*r* = −.26; *n* = 350; *p* < .0001), between ASBQ Total scores (parent-report) and IQ (*r* = −.38; *n* = 94; *p* = .0002) and between RBS-R Total scores and IQ (*r* = −190; *n* = 340; *p* = .0003) were observed. Scores on the SRS-2 (*T* scores and raw scores for self-report), AQ (child, adolescent and adult version), SSP and CSBQ (parent-report)/ASBQ (self-report) were not significantly associated with level of intellectual functioning.

### Psychiatric symptom measures (analysed within the ASD participants only)

Due to limited availability of self-report data (TD: *n* = 14; ASD: *n* = 18), only parent-reported levels of ADHD symptoms were analysed. A large proportion of children with ASD (here defined as chronological age <17 years according to the ADHD symptom checklist) scored in the clinical range on the inattentiveness (51%) and hyperactivity/impulsivity ADHD domains (28%). In contrast, the number of adolescents and adults with ASD that met clinical cut-off on these measures was somewhat lower (inattentiveness 41%; hyperactivity/impulsivity 13%). Among participants with ASD, males scored significantly higher than females on the inattentiveness domain (*x* ^*2*^(1) = 4.73, *p* = .030, *d* = .22) and hyperactivity/impulsivity domain (*x* ^*2*^(1) = 3.99, *p* = .046, *d* = .22). There was also a significant effect of schedule on both the inattentiveness domain (*x* ^*2*^(3) = 26.30, *p* < .0001) and hyperactivity/impulsivity domain (*x* ^*2*^(3) = 71.73, *p* < .0001), with adults with ASD having significantly lower symptom levels across these domains compared to children (inattentiveness: *x* ^*2*^(1) = 20.72, *p* < .0001, *d* = .78; hyperactivity/impulsivity: *x* ^*2*^(1) = 69.35, *p* < .0001, *d* = 1.32) and adolescents (inattentiveness: *x* ^*2*^(1) = 14.94, *p* = .0003, *d* = .54; hyperactivity/impulsivity: *x* ^*2*^(1) = 11.80, *p* = .002, *d* = .50). However, while no differences were observed between children and adolescents in inattentive symptom levels (*x* ^*2*^(1) = 0.60, *p* = .438), children with ASD had significantly higher levels of hyperactivity/impulsivity symptoms compared to adolescents with ASD (*x* ^*2*^(1) = 24.98, *p* < .0001, *d* = .87). There was no significant interaction effect between sex and schedule.

Among participants with ASD completing the BAI or BYI-II as self-report, 24% of adults (26 of 108; i.e. raw anxiety scores 21+) and 18% of adolescents (12 of 66; sex-and age-adjusted *T* score 60+) scored in the moderate/severe clinical range. In children (TD: *n* = 51; ASD: *n* = 83) and adolescents/adults with mild ID (mild ID: *n* = 10; ASD: *n* = 29), symptoms of anxiety were assessed by the BYI-II through parent-report. In addition, some adolescents without ID (TD: *n* = 4; ASD: *n* = 17) received the BYI-II as parent-report. The proportion of individuals with ASD considered to present with a moderate/severe severity level in anxiety symptoms (same clinical cut-offs apply as above) was 12% for children (10 of 83), 7% for adolescents (2 of 29) and 27% for adolescents/adults with mild ID (4 of 15). No significant effects of sex or schedule were found across all anxiety scales.

For depressive symptoms as measured by the BDI-II or BYI-II as self-report, it was found that among participants with ASD, 22% of adults (24 of 107; raw depression scores of 21+) and 27% of adolescents (18 of 67; i.e. *T* score 60+) scored in the moderate to severe clinical range. In adults with ASD, females reported significantly higher depressive symptoms than males (*x* ^*2*^(1) = 11.66, *p* = .0006, *d* = .72) but not in adolescents (*x* ^*2*^(1) = .44, *p* = .507). The depression subscale of the BYI-II was administered to children (TD: *n* = 53; ASD: *n* = 86), adolescents/adults with mild ID (mild ID: *n* = 10; ASD: *n* = 29) and adolescents without ID (TD: *n* = 4; ASD: *n* = 17) and completed by their parents. Sixteen percent of children (14 of 86), 29% of adolescents (5 of 17) and 28% of adolescents/adults with mild ID (8 of 29) had scores in the moderate/severe clinical range (i.e. sex- and age-adjusted *T* score of 60+).

### Association between psychiatric symptoms and intellectual functioning

Among participants with ASD, the association between psychiatric symptoms (depression, anxiety, inattention and hyperactivity/impulsivity) and intellectual functioning (full-scale IQ) was also assessed. There were significant but weak negative correlations between parent-reported symptoms of inattention and IQ (*r* = −.20; *n* = 345; *p* < .0001), as well as between hyperactivity/impulsivity and IQ (*r* = −.17; *n* = 345; *p* = .001). On measures of anxiety, no significant correlation was found between self-report measures and IQ in adolescents (*r* = −.10; *n* = 66; *p* = .421), as well as between parent-report measures and IQ in children, adolescents and adolescents/adults with mild ID (*r* = −.05; *n* = 125; *p* = .555). There was however a significant, albeit weak negative correlation between anxiety symptoms (self-report) and IQ in adults with ASD (*r* = −.23; *n* = 108; *p* = .017). No significant association between depressive symptoms (parent- or self-report) and IQ was observed across all schedules (all *p* > 0.1).

### Associations between ASD measures

**Fig. 4:**
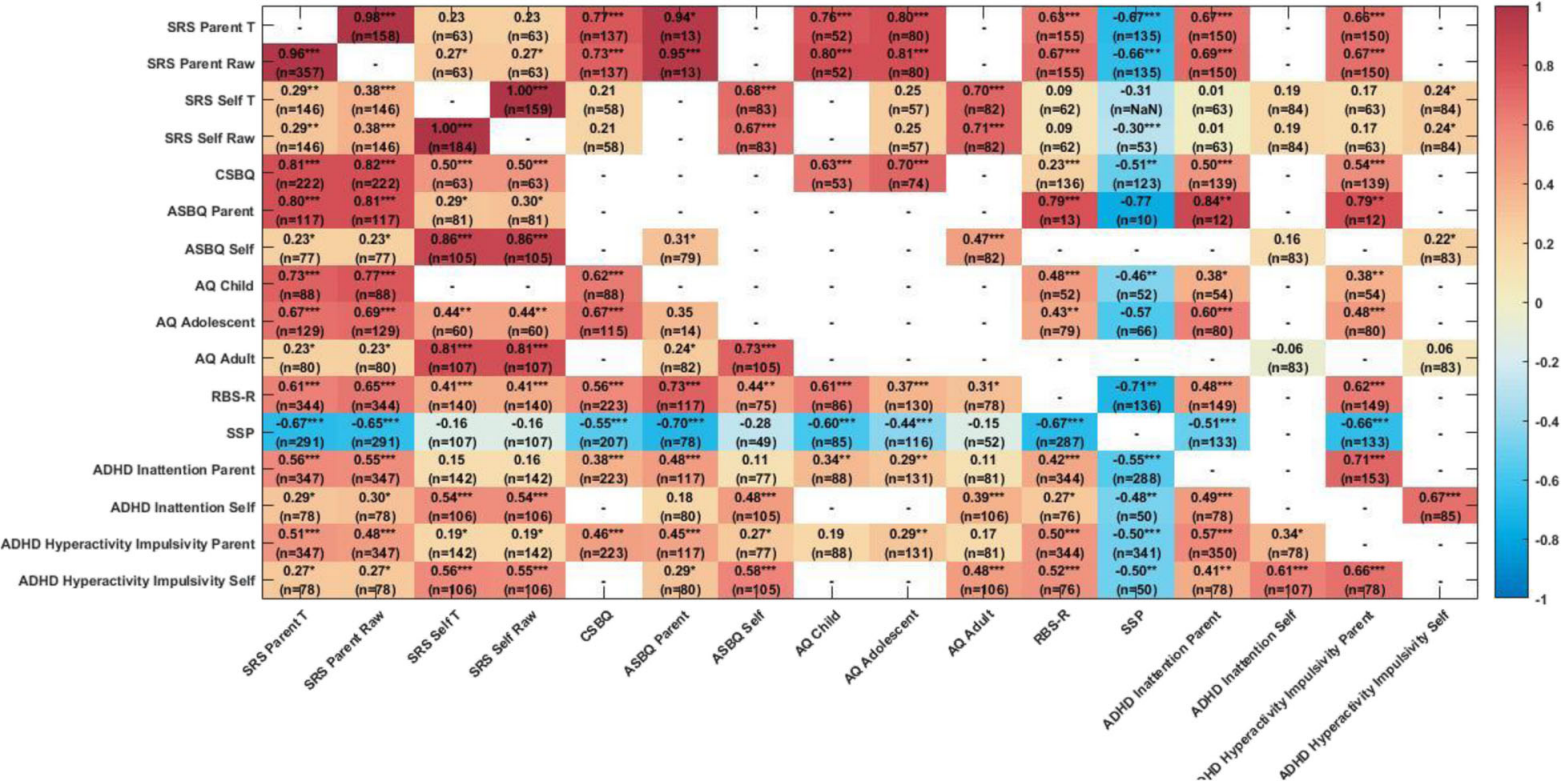
Heatmap of correlations between ASD and psychiatric symptom measures (ASD *left diagonal*; TD/ID participants *right diagonal*)

Figure 4 shows the associations between the different questionnaire ASD symptom measures separately for the ASD and TD/ID participants. Within the ASD group, as expected, the parent-report global ASD symptom measures (SRS, CSBQ,/ASBQ, AQ) were highly inter-correlated (all *r* values >.60, *p* < .0001). The RBS-R measuring repetitive behaviour symptoms (*r* from .56 to .73, all *p* < .0001) and the SSP measuring sensory symptoms (higher scores on the SSP indicate lower symptomatology; *r* from −.44 to −.70, all *p* < .0001) were also strongly inter-correlated with the global symptom measures. Parent-report of ASD symptoms (SRS, CSBQ/ASBQ) was moderately to strongly associated with parent-report of both ADHD inattention and hyperactivity/impulsivity symptoms (all *r* > .38, *p* < .0001) but the parent-report AQ less so (see Fig. 4).

## Discussion

### Clinical characteristics of the EU-AIMS LEAP cohort

The EU-AIMS LEAP cohort is a large, well-characterised sample of individuals with ASD and controls ranging from young children to adults with a fairly wide range of IQ. The main groups of adult, adolescent and child participants with ASD and controls have IQs in the typical range with means close to the population average. The group of purposively sampled participants with and without ASD with mild ID (IQ range 50 to 74) is relatively small (*n* = 68 ASD; *n* = 29 non-ASD). Although the LEAP sample has an elevated IQ compared to the total population of individuals with ASD, of whom around 50% have an intellectual disability [8, 9], it is rare for experimental studies of biomarkers to include any participants with an IQ below 75. Participants were purposively sampled to enable in depth experimental characterisation of potential biomarkers (including MRI scans), and therefore we set a lower IQ limit of 50; however, we enrolled 3 participants with lower IQ but who were capable of completing all our minimal assessments. It is a notable limitation of the representativeness of the current sample that in common with many studies, we excluded ASD participants with severe intellectual disability and this remains a challenge to scientific enquiry, in particular perhaps in the domain of cognitive neuroscience [84]. Related to this point, we note that the ADOS CSS scores were somewhat lower overall in the current LEAP sample (Table 7) compared to other large cohorts such as the Simons Simplex Collection [85] which predominantly consists of clinically ascertained samples and included participants with lower IQ than in the present volunteer research sample where IQ was restricted to IQ ≥50 due to the experimental protocol.

**Table 7:** Summary of ASD dimensional measures by schedule and group

	Total		Adults		Adolescents		Children		Mild ID	
	ASD	TD/ID	ASD	TD	ASD	TD	ASD	TD	ASD	ID
Autism symptomatology measures										
*SRS-2*	72.12^a^ (11.75) *n* = 357	47.96^a^ (9.64) *n* = 158	64.39^a^ (10.85) *n* = 99	47.59^b^ (5.89) *n* = 90	74.31^a^ (10.71) *n* = 105	45.71^a^ (6.38) *n* = 75	75.04^a^ (11.25) *n* = 90	44.86^a^ (5.28) *n* = 57	76.41^a^ (9.93) *n* = 63	64.55^a^ (10.29) *n* = 20
*CSBQ/ASBQ*	46.88^a^ (16.59) *n* = 225	7.10^a^ (9.94) *n* = 139	32.05^b^ (14.88) *n* = 102	7.85^b^ (8.65) *n* = 82	44.95^a^ (16.87) *n* = 105	5.88^a^ (8.71) *n* = 75	45.93^a^ (16.37) *n* = 87	5.21^a^ (7.91) *n* = 53	55.55^a^ (13.74) *n* = 33	24.55^a^ (10.61) *n* = 11
*AQ*	–	–	82.53 ^be^ (19.49) *n* = 105	47.26^be^ (14.92) *n* = 82	94.66^ad^ (17.92) *n* = 99	42.63^ad^ (16.49) *n* = 70	92.74^ac^ (17.18) *n* = 86	39.46^ac^ (17.15) *n* = 54	99.36^ad^ (17.33) *n* = 28	81.30^ad^ (12.02) *n* = 10
*RBS-R*	16.75^a^ (13.85) *n* = 346	2.52^a^ (8.04) *n* = 157	10.73^a^ (10.13) *n* = 91	–	16.53^a^ (13.95) *n* = 106	1.01^a^ (2.09) *n* = 73	19.21^a^ (12.94) *n* = 87	1.07^a^ (2.17) *n* = 56	22.55^a^ (16.82) *n* = 62	11.86^a^ (18.62) *n* = 22
*SSP*	138.13^a^ (27.46) *n* = 293	175.39^a^ (17.12) *n* = 141	155.62^a^ (23.88) *n* = 60	–	137.35^a^ (26.85) *n* = 96	180.52^a^(11.00) *n* = 62	130.31^a^ (24.77) *n* = 85	175.68^a^ (12.75) *n* = 56	132.17^a^ (28.37) *n* = 52	155.11^a^ (28.43) *n* = 19

*n*s are lower for some measures due to missing data

*SRS-2* Social Responsiveness Scale–2, *CSBQ/ASBQ* Children’s Social Behaviour Questionnaire (parent-report, administered to children and adolescents), Adults’ Social Behaviour Questionnaire (self-report, administered to adults), *RBS-R* Repetitive Behavior Scale–Revised, *SSP* Short Sensory Profile, *AQ* Autism Spectrum Quotient (children, adolescents or adult version)

^a^Parent-report

^b^Self-report

^c^AQ-Child

^d^AQ-Adolescent

^e^AQ-Adult

Reflecting recruitment from multiple research sites in four countries from existing research cohorts and from different clinic and volunteer sources, there were significant site effects on the core characterisation measures identified in the mixed-effects models. However, ICCs were mostly below 10% (the exception was age which reflects that some sites only sampled across some of the schedule groups). This reflects that there was considerable heterogeneity of cognitive ability levels and scores on core diagnostic measures within each site but systematic differences between sites on these measures ranged from minimal to moderate only. The quality control procedures we implemented give us confidence in the coherence and comparability of data collected across six sites.

In addition to the well-established diagnostic measures ADI-R and ADOS, we have further characterised ASD symptomatology using a range of dimensional parent-report (and, in adolescents and adults, self-report) measures of global ASD symptom severity (SRS-2, CSBQ/ASBQ, AQ) as well as specific measures of repetitive (RBS-R) and sensory (SSP) symptoms. Furthermore, we have also acquired questionnaire measures of the most commonly occurring psychiatric symptoms found in individuals with ASD [7, 40]—ADHD, anxiety and depression. In terms of the biomarker discovery aims of the EU-AIMS LEAP project overall [46–49], this comprehensive clinical characterisation of such a large sample will enable us to test for associations between putative biomarkers while including potential moderating or stratification factors including sex, age, IQ and co-occurring psychiatric symptoms.

### Sex differences in ASD symptoms

We examined sex differences in ASD severity that have been reported in some but not all previous studies [18]. Across the whole sample, males with ASD had more severe symptom scores than females on some domains of the ADOS and the ADI-R, including both social communication and repetitive behaviours. Some previous studies have found higher levels of repetitive behaviours but not higher social communication symptoms in males vs. females [19, 21], but others have reported higher levels of social communication symptoms in females [22, 23]. In contrast, we found no sex differences on the parent-report questionnaire measures of ASD symptoms (SRS-2, CSBQ/ASBQ, RBS-R and SSP). Diagnostic measures like the ADOS and ADI-R differ from the parent-report ASD symptom questionnaires in several ways, including that the ADOS is an observer-rated measure of current ASD symptoms and the ADI-R algorithm domain scores assess historical symptom severity (4 to-5 years and ever). The parent-report and self-report questionnaires by design are intended to measure symptoms or traits in a more continuous or dimensional fashion compared to these diagnostic tools. However, it remains unclear as to why males had higher ASD symptom severity scores on the diagnostic measures but not the questionnaire measures. One possible explanation is a bias or expectation of researchers administering the ADOS and ADI-R, perhaps due to expectations about sex differences—for example awareness of female compensatory behaviours and strengths—in ASD symptom profiles. Another possibility is that parent-reported questionnaire measures are influenced by parents’ gender stereotypes. Alternatively, diagnostic measures that tap variation in clinical level symptoms and ‘trait’ measures of individual differences across populations of the ASD phenotype are of a different kind, although recent twin studies suggest that they share a common genetic architecture [86]. A final point to note is that, with the notable exception of the SRS-2, none of the other measures have sex-specific norms which should be a future goal for further psychometric development of ASD symptom measures (Table 8) [18, 87].

**Table 8:** Summary of psychiatric symptom measures by schedule and group

	Total		Adults		Adolescents		Children		Mild ID	
	ASD	TD/ID	ASD	TD	ASD	TD	ASD	TD	ASD	ID
Psychiatric symptom measures										
*ADHD—inattentiveness*	4.55^a^ (3.15) *n* = 350	1.29^a^ (2.34) *n* = 153	3.18^a^ (3.19) *n* = 94	0.88^b^ (1.67) *n* = 84	4.82^a^ (3.19) *n* = 106	0.88^a^ (1.79) *n* = 75	5.20^a^ (2.98) *n* = 88	0.67^a^ (1.60) *n* = 54	5.21^a^ (2.67) *n* = 62	5.17^a^ (2.85) *n* = 18
*ADHD—hyperactivity/impulsivity*	2.83^a^ (2.86) *n* = 350	0.56^a^ (1.59) *n* = 153	1.22^a^ (1.72) *n* = 94	0.55^b^ (1.32) *n* = 84	2.70^a^ (2.78) *n* = 106	0.19^a^ (0.82) *n* = 75	4.41^a^ (2.93) *n* = 88	0.35^a^ (1.15) *n* = 54	3.27^a^ (2.99) *n* = 62	2.94^a^ (2.94) *n* = 18
*Anxiety*	–	–	15.05^bc^ (12.76) *n* = 104	4.41^bc^ (5.19) *n* = 85	49.92^bd^ (10.63) *n* = 65	44.42^bd^ (7.55) *n* = 65	47.81^ad^ (8.96) *n* = 83	38.33^ad^ (5.18) *n* = 51	49.17^ad^ (9.45) *n* = 29	45.40^ad^ (12.45) *n* = 10
*Depression*	–	–	14.11^bc^ (12.41) *n* = 103	3.88^bc^ (4.93) *n* = 85	51.48^bd^ (10.55) *n* = 66	45.56^bd^ (8.05) *n* = 66	49.98^ad^ (10.22) *n* = 86	39.29^ad^ (4.71) *n* = 56	53.17^ad^ (10.07) *n* = 29	50.20^ad^ (18.01) *n* = 10

*n*s are lower for some measures due to missing data

*ADHD—inattentiveness* (ADHD rating scale—inattentiveness subscale), *ADHD—hyperactivity/impulsivity* (ADHD rating scale—hyperactivity/impulsivity subscale), *Anxiety* Beck Anxiety Inventory, Depression (Beck Depression Inventory–Second Edition)

^a^Parent-report

^b^Self-report

^c^Raw scores

^d^Standardised scores

### Age and IQ differences in ASD symptoms

On the diagnostic measures (ADOS and ADI-R), there were no age differences in symptom severity. However, on the SRS-2 (a parent-report global measure of ASD symptoms), adults with ASD had lower symptom severity than adolescents and children and the ASD group with mild ID. A similar pattern was found on the parent-report measure of restricted, repetitive and stereotyped behaviour, the RBS-R, with adults with ASD scoring lower than all other groups. The findings were corroborated when age was analysed in a continuous fashion rather than according to the age and ability schedule presented here (see Additional file 2: Table S1). This is consistent with a number of other studies showing reduced ASD symptoms in adulthood, including samples followed longitudinally since childhood [34–36]. With only one time-point of data, we cannot yet determine if the age differences in symptom severity are due to cross-sectional differences in sampling or true in nature but the accelerated longitudinal design of the LEAP study will allow us to investigate this in the future.

Social communication symptoms as measured by the ADOS Social Affect CSS and ADI-R Social and Communication domain scores were moderately negatively associated with IQ—with higher scores in those with lower IQ—but this was not the case for the ADOS RRB CSS or the ADI-R RRB domain. On the continuous measures of ASD symptomatology, the SRS-2 and RBS-R were also correlated negatively with IQ but the AQ and SSP were not. Note, however, that even when these associations were significant in this large and well-powered sample, the variance in common between IQ and symptom measures (*r*-squared) was only ~5%. This is in line with previous studies where low IQ has been modestly but significantly associated with higher levels of ASD symptom severity [41, 42]. This may, in part, reflect the fact that many diagnostic and dimensional measures of ASD symptomatology include a mixture of developmental abilities or skills and frank atypical behaviours, in particular for children and adolescents. Alternatively, individuals with ASD with higher cognitive ability might develop compensatory or alternative strategies to develop social communication skills resulting in slightly reduced symptom presentation. When looking at associations between putative ASD biomarkers and measures of the core ASD phenotype and co-occurring psychiatric symptoms, it will be important to consider the effect of IQ as associations dependent or independent of intellectual ability might indicate different neurobiological mechanisms.

### Co-occurring psychiatric symptoms

Among individuals with ASD, males had higher levels of inattentive and hyperactive/impulsive symptoms than females and both inattentive and hyperactive/impulsive symptoms were lower in adults than in adolescents, as has been found in non-ASD samples [88]. Female adults with ASD reported higher levels of depressive, but not anxiety, symptoms than males. This finding is potentially important to emphasise so that clinicians do not overlook possible symptoms of depression in adult females with ASD. The proportion of individuals with elevated anxiety scores is lower in the current sample than in many previous studies, but note that we were using questionnaire screening measures of psychiatric symptoms and not diagnostic instruments where 30 to 40% of individuals with ASD have met criteria for an anxiety disorder [7, 89]. Parent-report and self-report of co-occurring psychiatric symptoms were weakly negatively correlated with IQ, consistent with some previous studies [38, 45]. Most parent-report measures of ASD symptoms were moderately to strongly associated with parent-report of both ADHD inattention and hyperactivity/impulsivity symptoms [90] (and similarly for self-reported ASD symptoms and self-reported associated psychiatric symptoms) but the AQ somewhat less so (see Fig. 4). Parent-report of ASD symptoms was only moderately associated with self-reported anxiety and depression, as has been previously reported in ASD [91] and non-ASD samples [92]. We note that the validity of assessments of psychiatric symptoms in samples of individuals with ASD is unknown, perhaps especially with respect to anxiety symptoms, although the measures we chose are widely used, including in previous studies in ASD.

### Self-report measures of the ASD phenotype

In contrast to the higher symptom scores in males compared to females on the diagnostic measures the ADOS and ADI-R (but not on parent-report questionnaire measures of symptom severity), in a sub-sample of adults and adolescents with ASD able to self-report on the SRS-2, ASBQ and AQ female adults reported higher levels of symptoms than males. A similar pattern has been reported in previous studies [93, 94] and may be due to higher self-reflective ability in adult females than males with ASD, identity-driven ‘biases’ or truly heightened ASD traits. The different pattern of findings for self- vs. parent-report of ASD symptoms might also indicate an effect described as ‘masking’ or ‘camouflage’ in (adult and adolescent) females with ASD whereby symptoms appear ameliorated to observers (in this case parents) due to compensatory social engagement skills [18]. We also found contrasting patterns of self- vs. parent-report of ASD symptoms with respect to age, with parent report SRS-2 scores showing lower symptoms in adults than adolescents but self-report finding the reverse. One important contribution the current study makes is the inclusion of a range of ascertainment methods of ASD symptoms including clinician observation and both parent- and self-report. These are important considerations both for identifying biomarkers associated with the ASD phenotype and potentially for use as outcome measures in future clinical trials. The issues raised are complex and go beyond the sample description contained in the current paper but include what it might mean if biomarkers relate to one type of measure but not another and what measures (e.g. clinician-report vs. parent-report vs. self-report) should be used as outcome measures in clinical trials and who gets to make these choices [61].

### Relevance of in-depth clinical characterisation for biomarker analysis

Within the framework of the NIH Research Domain Criteria (RDoC; [95]) initiative, core neurobiological or genetic systems vulnerabilities might map better onto neurodevelopmental or neurocognitive systems than the disorder-specific behavioural domains. This guided the ‘deep phenotyping’ approach we have taken in the EU-AIMS LEAP study to characterise the cohort not only comprehensively in terms of their ASD and co-occurring disorders behavioural phenotype but also at the level of structural and functional brain development, neurocognitive function and biochemical and genomic assays [46], consistent with other ‘big data’ approaches in psychiatry [17].

Choices as to *which* ASD symptom measures should be used for biomarker validation need to be informed by a number of considerations. These include statistically guided principles regarding distributions (in both cases and controls). Across the range of ASD phenotypic measures acquired in the LEAP sample, some are highly skewed even in the ASD sample (e.g. SSP), while other measures are dimensional and more akin to ‘trait’ measures and have considerable variation in both the ASD and control samples (e.g. SRS-2, CSBQ/ASBQ, AQ). Although skewed data can be statistically transformed back towards normality, non-parametric, ordinal or categorical approaches can also be adopted but this needs to be mapped back onto the clinical phenomena that any phenotypic measure is assaying. Another consideration will be the extent to which potential biomarkers are examined in terms of their association with ‘domains’ or ‘sub-domains’ of the ASD phenotype, for example within the repetitive behaviours domain there is some evidence at the genetic level that different genes might associated with ‘lower’ vs. ‘higher’ levels of repetitive behaviour [96]. Finally, we have reported both raw and age and sex-normed *T* scores on an instrument such as the SRS-2 in this clinical paper but for biomarker analysis raw un-adjusted scores allows a more neutral mapping onto the phenotypic behaviour.

The diagnostic measures have particular characteristics that might make them useful at different levels/stages in the biomarker validation process. For example, the ADI-R diagnostic algorithm domain scores are based on past history and in particular the early developmental period (age 4 to 5 years) when it has been proposed that ASD presentation is most prototypical [97]. On the other hand, the ADOS is a researcher/clinician-rated observational measure and is therefore less likely to suffer from the same potential ‘halo effect’ when a parent is rating (for example, on two questionnaires) different behavioural characteristics (e.g. ASD and ADHD), thus reducing systematic rater bias.

We have also found modest but robust associations between severity of ASD symptoms and participant characteristics such as age, sex and IQ as well as with levels of co-occurring psychiatric symptoms. These considerations will be important for considering the sensitivity and specificity of any associations found between the ASD phenotype and potential biomarkers. The associations between potential stratification biomarkers and ASD symptoms can be tested in models that include these factors where they are associated with the ASD phenotypic scores themselves. The LEAP cohort has purposively been ‘deep phenotyped’ at a number of levels so that biomarker detection analysis in this large sample can take account of these factors.

## Conclusions

The in-depth clinical characterisation of the EU-AIMS LEAP cohort will allow us to test how a wide range of potential biological and neurocognitive biomarkers [46–49] are associated with both diagnostic and more dimensional measures of the core ASD phenotype. We will be able to test whether these associations are influenced by the presence of commonly co-occurring psychiatric symptoms, as well as whether they differ across males and females or according to age or intellectual ability. In addition, the pattern of associations we have found in the LEAP cohort differs across the clinician observational and parent- vs. self-report questionnaire measures and both conceptual and methodological considerations should guide how these issues are addressed in stratification biomarker analysis. The inclusion of multiple dimensional measures of ASD symptom severity will allow us to test which measure relates best to neurobiological or neurocognitive biomarkers and is most sensitive to change over time. This would have important implications for choosing appropriate outcome measures in future clinical trials. We anticipate that as the EU-AIMS LEAP cohort is followed into the future, it will become a key resource of autism discovery science.

## Additional files

Additional file 1: Table S3. (DOCX 22 kb)

Additional file 2: Table S1. Predicted effect of age and IQ on ADOS Calibrated Severity Scores. (DOCX kb)

Additional file 3: Table S2. Predicted effect of age and IQ on parent-and self-report ASD measures. (DOCX kb)

## Abbreviations

ADHD: Attention-deficit/hyperactivity disorder
ADI-R: Autism Diagnostic Interview-Revised
ADOS: Autism Diagnostic Observation Schedule
AQ: Autism Spectrum Quotient
ASBQ: Adult Social Behaviour Questionnaire
ASD: Autism Spectrum Disorder
BAI: Beck Anxiety Inventory
BDI-II: Beck Depression Inventory—Second Edition
BYI-II: Beck Youth Inventories
CIMH: Central Institute of Mental Health
CSBQ: Children’s Social Behaviour Questionnaire
CSS: Calibrated Severity Scores
DSM-5: Diagnostic and Statistical Manual of Mental Disorders, Fifth Edition
DSM-IV: Diagnostic and Statistical Manual of Mental Disorders, Fourth Edition
DSM-IV-TR: Diagnostic and Statistical Manual of Mental Disorders, Fourth Edition text-revision
EU-AIMS: European Autism Interventions—A Multicentre Study for Developing New Medications
ICC: Intraclass correlation coefficient
ICD-10: International Statistical Classification of Diseases and Related Health Problems, 10th Revision
ID: Intellectual disability
IoPPN: Institute of Psychiatry, Psychology and Neuroscience
IQ: Intelligent quotient
KCL: King’s College London
LEAP: Longitudinal European Autism Project
MRI: Magnetic resonance imaging
P: Parent-report
RBS-R: Repetitive Behavior Scale-Revised
RDoC: Research Domain Criteria
RRB: Repetitive and Restricted Behaviours
RUNMC: Radboud University Nijmegen Medical Centre
S: Self-report
SA: Social Affect
SRS-2: Social Responsiveness Scale, Second Edition
SSP: Short Sensory Profile
TD: Typically developing
UCAM: Autism Research Centre, University of Cambridge
UCBM: University Campus Bio-Medico
UMCU: University Medical Centre Utrecht
WAIS-III/IV: Wechsler Adult Intelligence Scale—Third Edition/Fourth Edition
WASI-II: Wechsler Abbreviated Scales of Intelligence—Second Edition
WISC-III/IV: Wechsler Intelligence Scale for Children—Third Edition/Fourth Edition

## Footnotes

1 At four additional sites (UCAM, CIMH, UCBM and UMCU) following assessment, a minority of participants with ASD were allocated to the Mild ID group due to measured IQ falling in the 50–74 range (see Table 1).

2 There are three individuals with a Full-scale IQ <50 in the sample (all ASD).
