# Supplementary material for: The EU-AIMS Longitudinal European Autism Project (LEAP): clinical characterisation
Source: Mol Autism. 2017 Jun 23;8:27. doi: 10.1186/s13229-017-0145-9 (PMC5481972; doi:10.1186/s13229-017-0145-9)
Supplement: Supplementary file 1 — (DOCX 22 kb) [file 13229_2017_145_MOESM1_ESM.docx]

Supplementary Table 3

| **Domain/ Task** | **A**  **(TD)** | **A**  **(ASD)** | **B**  **(TD/ASD)** | **C (TD/ASD)** | **D**  **(ID/ASD)** |
| --- | --- | --- | --- | --- | --- |
| **Clinical Diagnosis** |  |  |  |  |  |
| - Autism Diagnostic Interview-Revised (ADI-R) | - | P^a^ | P^a^ | P^a^ | P^a^ |
| - Autism Diagnostic Observation Schedule (ADOS or ADOS-2) | - | S^a^ | S^a^ | S^a^ | S^a^ |
| **Dimensional measures of ASD symptoms** |  |  |  |  |  |
| - Social Responsiveness Scale-2^nd^ Edition (SRS) | S | S & P | S & P | P | P |
| - Repetitive Behaviour Scale-Revised (RBS-R) | - | P | P | P | P |
| - Short Sensory Profile (SSP) | - | P | P | P | P |
| - Children’s Social Behaviour Questionnaire (CSBQ) - Adults’ Social Behaviour Questionnaire (ASBQ) | -  S | -  S & P | P  - | P  - | P  P(>18yrs) |
| - Autism Quotient (AQ), AQ-Adol, AQ-Child | S | S & P | P | P | P |
| - Aberrant Behaviour Checklist | - | P | P^%^ | P^%^ | P |
| - Adult Routine Inventory (ARI) - Child Routine Inventory (CRI-R) | S | S | P | P | P |
| - Sensory Experiences Questionnaire - short version(SEQ 3.0) | - | - | P | P | P |
| - Global Score of Change | - | P | P^a^ | P ^a^ | P^a^ |
| **Comorbidities** |  |  |  |  |  |
| - Development and Well-Being Assessment (DAWBA) | - | S & P | S & P | P | P |
| - Strengths and Difficulties Questionnaire (SDQ), | S | S & P | S & P | P | P |
| - DSM-5 ADHD rating scale | S | S & P | P | P | P |
| - Beck Anxiety Inventory | S | S | S | P | P |
| - Beck Depression Inventory | S | S | S | P | P |
| **Quality of Life/Adaptive Behaviour** |  |  |  |  |  |
| - Vineland-II Adaptive Behaviour Scale | - | P | P | P | P |
| - Columbia impairment scale (CIS) | S | S & P | S & P | P | P |
| - Child-Health and Illness Profile (CHIP-CE), or - WHOQOL-BREF | -  S | -  S | P  - | P  - | P  - |
| **Medical/ psychiatric history** |  |  |  |  |  |
| - NIH ACE Subject Medical History Questionnaire | S | P | P | P | P |
| - NIH ACE Family History Form | S | P | P | P | P |
| - Peri/Pre-natal environmental questionnaire | S | P | P | P | P |
| - Children’s Sleep Habits Questionnaire - Adult version FU only | S | S | S & P | P | P |

Note: P = reported by parent; S= self-reported; TD = typical development; ASD = Autism Spectrum Disorder; ID = Intellectual Disability; Schedule A: Adults with ASD or TD (aged 18-30 years, with IQ greater than 75); Schedule B: Adolescents with ASD or TD (aged 12-17 years, with IQ greater than 75); Schedule C: children with ASD or TD (aged 6-11 years, with IQ greater than 75); Schedule D: Adolescents and adults with mild ID (with or without ASD) (aged 12-30 years, with IQ 50-75);
^a^ ASD groups only
