# Supplementary material for: The EU-AIMS Longitudinal European Autism Project (LEAP): clinical characterisation
Source: Mol Autism. 2017 Jun 23;8:27. doi: 10.1186/s13229-017-0145-9 (PMC5481972; doi:10.1186/s13229-017-0145-9)
Supplement: Supplementary file 2 — Predicted effect of age and IQ on ADOS Calibrated Severity Scores. (DOCX kb) [file 13229_2017_145_MOESM2_ESM.docx]

Supplementary Table 1 Predicted effect of age and IQ on ADOS Calibrated Severity Scores

|  | Chronological age | | | | |  | Intellectual functioning | | | | |
| --- | --- | --- | --- | --- | --- | --- | --- | --- | --- | --- | --- |
| Variable | b | SE(b) | t | p-value | 95% CI |  | b | SE(b) | t | p-value | 95% CI |
| ADOS – CSS Total | -.022 | .025 | 0.89 | .375 | [-.07, .03] |  | -.033 | .007 | 4.39 | < .001 | [-.05, -.02] |
| ADOS – CSS  SA | -.007 | .024 | 0.28 | .777 | [-.05, .04] |  | -.033 | .007 | 4.69 | < .001 | [-.05, -.02] |
| ADOS – CSS RRB^a^ | -.001 | .007 | 0.17 | .864 | [-.02, .01] |  | -.002 | .002 | 0.95 | .340 | [.91, 2.09] |

Note: b = regression coefficient, SE(b) = standard error of regression coefficient, t = t-statistic, 92% CI = 95% Confidence Interval of regression coefficient
*ADOS CSS Total, SA, RRB* = Autism Diagnostic Observation Schedule Calibrated Severity Scores for Total, Social Affect and Restricted and Repetitive Behaviours

^a^ log-transformed
