# Supplementary material for: The EU-AIMS Longitudinal European Autism Project (LEAP): clinical characterisation
Source: Mol Autism. 2017 Jun 23;8:27. doi: 10.1186/s13229-017-0145-9 (PMC5481972; doi:10.1186/s13229-017-0145-9)
Supplement: Supplementary file 3 — Predicted effect of age and IQ on parent-and self-report ASD measures. (DOCX kb) [file 13229_2017_145_MOESM3_ESM.docx]

Supplementary Table 2 Predicted effect of age and IQ on parent-and self-report ASD measures

|  | Chronological age | | | | |  | Intellectual functioning | | | | |
| --- | --- | --- | --- | --- | --- | --- | --- | --- | --- | --- | --- |
| Variable | b | SE(b) | T | p-value | 95% CI |  | b | SE(b) | t | p-value | 95% CI |
| **Parent-report (all age groups)** | | | | | | | | | | | |
| SRS-2 T-score^a^ | -.815 | .098 | 8.31 | < .001 | [-1.01, -.62] |  | -.160 | .027 | 5.84 | < .001 | [-.21, -.11] |
| SRS-2 Raw^a^ | .234 | .326 | 0.72 | .472 | [-.40, .87] |  | -.692 | .086 | 8.04 | < .001 | [-.86, -52] |
| RBS-R^ac^ | -.050 | .009 | 5.99 | < .001 | [-.02, -.01] |  | -.010 | .001 | 4.42 | < .001 | [-.02, -.01] |
| SSP^ac^ | .01 | .010 | 6.15 | < .001 | [.01, .02] |  | .010 | .001 | 3.05 | .002 | [.01, .02] |
| **Parent-report (children & adolescents only)** | | | | | | | | | | | |
| CSBQ^a^ | -.397 | .380 | 1.05 | .296 | [-1.14, .35] |  | -.164 | .060 | 2.74 | .006 | [-.28, -.05] |
| **Parent-report (children-only)** | | | | | | | | | | | |
| AQ^a^ | 1.94 | 1.20 | 1.62 | .106 | [-.41, 4.29] |  | .215 | .116 | 1.85 | .064 | [-.01, 44] |
| **Parent-report (adolescents-only)** | | | | | | | | | | | |
| AQ^a^ | .780 | .541 | 1.44 | .149 | [-.28, 1.84] |  | .127 | .078 | 1.62 | .105 | [-.03, .28] |
| **Parent-report (adults-only)** | | | | | | | | | | | |
| ASBQ^a^ | .032 | .378 | 0.09 | .931 | [-.71, .77] |  | -.393 | .058 | 6.81 | < .001 | [-.51, -.28] |
| **Self-report (adults-only)** | | | | | | | | | | | |
| SRS-2 T-score^b^ | .344 | .155 | 2.22 | .027 | [.04, .65] |  | -.026 | .047 | 0.55 | .585 | [-.12, .08] |
| SRS-2 Raw^b^ | .289 | .382 | 0.75 | .450 | [-.46, 1.04] |  | -.372 | .121 | 3.07 | .002 | [-.61, -.13] |
| ASBQ^b^ | 1.03 | .418 | 2.46 | .014 | [.21, 1.85] |  | -.180 | .093 | 1.98 | .048 | [-.37, -.01] |
| AQ^b^ | 1.42 | .512 | 2.77 | .006 | [.41, 2.42] |  | -.057 | .11 | 0.51 | .613 | [-.28, .16] |

Note: b = regression coefficient, SE(b) = standard error of regression coefficient, t = t-statistic, 92% CI = 95% Confidence Interval of regression coefficient

*SRS-2* = Social Responsiveness Scale – 2; *CSBQ, ASBQ* = Children’s Social Behaviour Questionnaire (administered to children and adolescents), Adults’ Social Behaviour Questionnaire (administered to adults); *RBS-R* = Repetitive Behavior Scale – Revised; *SSP* = Short Sensory Profile; *AQ* = Autism Spectrum Quotient (children, adolescents or adult version)

^a^ Parent-report; ^b^ Self-report; ^c^ log-transformed

ps only
